# Anatomic variations of the Uterine Artery. Review of the literature and their clinical significance

**DOI:** 10.4274/tjod.galenos.2020.33427

**Published:** 2020-04-06

**Authors:** Konstantinos Liapis, Nikolaos Tasis, Ioannis Tsouknidas, George Tsakotos, Panagiotis Skandalakis, Konstantinos Vlasis, Dimitrios Filippou

**Affiliations:** 1 National and Kapodestrian University of Athens Medical School, Department of Anatomy and Surgical Anatomy, Athens, Greece

**Keywords:** Uterine artery, branch, origin, anatomic variation

## Abstract

Uterine arteries are the main vessels supplying blood to the uterus. Mainly, they originate from the anterior trunk of the internal iliac artery. Uterine arteries play an important role in pregnancy as well as transcatheter arterial embolization for postpartum hemorrhage and uterine fibroid management. This is a review of the English literature in the PubMed database of the anatomic variety on the origin of uterine arteries and their clinical significance. Eleven studies describe the origin of the uterine arteries and their variations in the literature. In six studies, the uterine artery emerged from internal iliac artery in the majority of the cases, either as a separate branch, or as a bifurcation with the inferior gluteal artery, or trifurcation with superior and inferior gluteal artery. In two studies, the inferior gluteal artery manifested as the main source of the uterine artery, whereas in three studies, the umbilical artery posed as its main origin. Internal iliac artery is described as the most common vascular origin of uterine artery. However, this review highlights that the main vessels of origin for uterine arteries are internal iliac, umbilical and inferior gluteal artery. Nevertheless, classification and further research for this peculiar anatomic structure is fundamental in the future.

## Introduction

Uterine arteries are the main vessels supplying blood to the uterus. They impose a significant clinical role in multiple medical conditions, especially during pelvic and gynecologic surgery. Uterine arteries go through dramatic changes during pregnancy^(1)^, increasing in volume and becoming more tortuous, playing an important role in perinatal outcomes^(2)^. Furthermore, uterine arteries are the target of embolization when dealing with fibroids and leiomyomata^(3)^ as well as uterine bleeding^(4)^, either because of pathologies mentioned or postpartum hemorrhage^(5)^. Uterine artery location and origin is also important in pelvic surgery. High vascular ligation of the afore-mentioned artery is a necessary step during hysterectomy, myomectomy, and other and gynecologic oncology procedures^(6)^. However, uterine arteries demonstrate a plethora of anatomic variation mainly concerning their origin, raising a challenge for the surgeon. This is a review of the literature about uterine artery anatomic variations and their clinical value.

### Clinical anatomy of uterine artery

The uterine artery traditionally arises from the internal iliac artery, anteriorly^(7)^. Partly the uterine artery passes, medially, through the base of the broad ligament of uterus before bifurcating at the isthmus level^(8)^. The ascending branch travels in parallel along the side of the uterus and fallopian tubes, following a U path and gives coil-shaped branches called the helicine branches. The ascending branch of the uterine artery anastomoses to the ovarian artery^(9)^. The descending part supplies the cervix and vagina^(10)^, anastomosing with the vaginal arteries and the inferior rectal arteries^(11)^. The uterine artery crosses the ureter superiorly at the level of the lateral part of the uterine cervix below the isthmic part of the uterus, explaining why the ureter is at greater risk of injury during pelvic and gynecologic surgeries^(12)^.

For the current paper, we reviewed the English literature in the PubMed database for articles concerning variations in uterine artery anatomy, spanning years 1900 to 2019. The search included the keywords uterine artery or internal iliac artery or hypogastric artery and anatomy or anatomic variations. We revised the results maintaining only cadaveric, surgical and radiologic studies on the anatomy of the uterine arteries and their variations. We also went through the references of the papers found, in order to discover more bibliographic resources.

Eleven studies describe the origin of the uterine artery and its variations in the literature. Article chronology ranges from 1918^(13)^ to 2019^(14)^, with more than half after 2010, indicating the increasing need for deeper understanding of uterine blood supply that has arisen after consolidation of uterine artery embolization for several uterine pathologies. Four studies were cadaveric^(13,15,16,17)^ and five studies were radiological, four of which used computed tomography (CT) angiography with 3D reconstruction^(14,18,19,20)^ and the fifth one included an evaluation of angiographies during uterine fibroid embolization^(21)^. One study uses surgical evaluation^(22)^ and one study combines all methods^(23)^. The combined results on the origin of uterine artery in our review are described in Table 1.

In six studies, the uterine artery emerged from internal iliac artery in the majority of their cases, either as a separate branch or as a bifurcation with inferior gluteal artery or trifurcation with superior and inferior gluteal artery. Kozlov et al.^(15)^ presented the internal iliac artery as the origin of the uterine artery in 60% of cases, followed by the umbilical artery as a separate branch, and via a common trunk in 27% and 1.8%, respectively. Albulescu et al.^(19)^ described the origin of the uterine artery as a separate branch from the internal iliac, as a bifurcation with the inferior gluteal, and as a trifurcation with superior and inferior gluteal arteries in 37%, 10%, and 29%, correspondingly. The remaining 24% arose from the inferior gluteal artery as a separate branch. Obimbo et al.^(16)^ demonstrated the uterine artery as the first branch of the internal iliac artery in 18.9% and second or third branch in 70.8% of cases. They also analyzed the course of uterine arteries in relation to the ureter and their branches. In 96.2% (102/106) of cases, the artery passed the ureter anteriorly, and in 3.8% (4/106 cases) it passed posteriorly. The uterine artery continues as a single vessel, bifurcates upon reaching the uterus or trifurcates in 76.4% (81/106), 17.1% (18/106), and 6.7% (7/106), respectively. Naguib et al.^(20)^ reported the uterine artery as a branch of the anterior division of internal iliac artery in 90% (86/95) of cases, as a branch of the posterior, main stem or bifurcation of internal iliac artery in 1%, 2%, and 1%, respectively. The uterine artery emerges via a common trunk with the obturator and internal pudendal in 5% and 1%, as well in this study. Roberts et al.^(17)^ described the uterine artery as a separate branch of the internal iliac, as a branch of the umbilical, inferior vesical, and internal pudendal in 27, 19, one, and one cases, respectively. Lipshutz^(13)^ also posed the internal iliac artery as the main origin of the uterine artery in 60 out of 67 cases, with the remaining four, two, and one originating from the superior vesical, internal pudendal, and obturator arteries, respectively.

In two studies, the inferior gluteal artery manifested as the main source of the uterine artery. Yunxiu et al.^(14)^ reported the uterine artery as a separate branch of the inferior gluteal artery in 64.3% (144/224) of cases. The uterine artery emerged independently from the internal iliac and via its trifurcation in 22.8% (51/224) of cases and 12.9% (29/224) of cases, respectively, in this study. Gomez-Jorge et al.^(21)^ described the uterine artery as a branch of the inferior gluteal, internal iliac artery or as a trifurcation with inferior and superior gluteal arteries in 51%, 43%, and 6%, respectively.

In three studies, the umbilical artery posed as the main origin of the uterine artery. Arfi et al.^(18)^ reported the uterine artery arose via a common trunk with the umbilical artery in 62.4% (54/86) of cases. In 25.6% (22/86) of cases, it was a branch of the internal iliac artery, in 9.3% (8/86) a branch of the superior gluteal artery, and in 2.3% (2/86) of cases a branch of the internal pudendal. Chantalat et al.^(23)^ also described the origin of the uterine artery via a common trunk with the umbilical artery in the majority of the cases in cadaveric, surgical, and radiologic groups. In the cadaveric group, 50 out of 60 (83.3%) uterine arteries emerged via a common trunk with the umbilical, six (10%) as a separate branch of the internal iliac, and four (6.7%) as a branch of the internal pudendal artery. In the surgical group, a common trunk of the umbilical and uterine arteries was noted in 82 cases (82%), 16 uterine arteries presented as independent branches of the internal iliac, and two of the superior gluteal arteries. In the angiography group, a common trunk of the uterine and umbilical arteries was described in 42 out of 58 cases (76.5%). The uterine artery was a branch of the internal iliac, superior gluteal, and obturator arteries in eight (11.8%), six (8.8%), and two (2.9%) cases, respectively. Finally, Holub et al.^(22)^ described the uterine artery as a branch of the umbilical and internal pudendal arteries in 76.5% and 23.5% of cases, respectively.

The uterine artery is an artery found in females that anatomically corresponds to the artery to the ductus deferens in males. Traditionally, it originates from internal iliac artery. However, following our results, the uterine artery does not always follow the typical route and origin. Except for the anterior trunk of the internal iliac, the uterine artery may originate from the inferior gluteal and umbilical artery, either directly or as a common stem. As reported in this review, these variations are quite frequent. There are rare cases in which the uterine artery comes from other arteries such as the superior gluteal, internal pudendal, obturator or vesical arteries. Moreover, there are some case reports of uncommon anatomic variations, such as the origin of uterine artery being from external iliac artery^(24)^ and inferior epigastric artery^(25)^. The absence of uterine arteries has also been observed^(26,27)^ with large ovarian vessels being present taking up the uterus blood supply.

The uterine artery and its high vascular ligation is a vital step during pelvic and gynecologic surgeries, such as hysterectomy. Often, pelvic pathologies such as fibroids, endometriosis, adhesions from previous pelvic surgeries, or ovarian remnants can distort the anatomic relations and create technical challenges during laparoscopic hysterectomies. Retroperitoneal dissection, in order to ligate the uterine artery at its vascular origin, can circumvent these obstacles, resulting in a safer procedure^(6)^.

Uterine artery anatomy and flow play an important role during pregnancy. The uterine artery becomes more tortuous, large, and with increased flow in pregnant women^(28)^. Ultrasound is a useful tool in the evaluation of the uterine artery during the first and second trimesters of pregnancy, most commonly used as uterine Doppler ultrasound, for the prediction of the later development of pre-eclampsia, intrauterine fetal growth restriction, placental abruption, and stillbirth^(2)^.

Furthermore, the clinical importance of understanding the uterine artery variations lies in the fact that during specific clinical processes, such as in uterine artery embolization, the plethora of anatomic variations of the uterine artery make the associate procedures quite challenging. As first described by O’Leary et al.^(29)^, one method of controlling postpartum hemorrhage is by bilateral ligation of the uterine arteries. Other methods include bimanual compression, which can be internal bimanual uterine compression or external bimanual compression or a medical approach, which includes oxytocin, tranexamic acid, blood transfusions or oral misoprostol. Occlusion of uterine vessels can be achieved via transcatheter arterial embolization, which is nowadays considered as the first-line therapy to control post-partum hemorrhage due to its characteristics of fast pace, excellent effect, wide indication, minimal invasiveness, and uterine preservation^(30)^. Embolization makes it possible to avoid hysterectomy, while theoretically preserving the possibility of another pregnancy^(5)^. Cheng et al.^(31)^ described how menstruation and fertility could be preserved successfully for future pregnancy after embolization.

Uterine artery embolization illustrates an important role in uterine fibroid and leiomyoma management. Embolization of the uterine arteries reduces the blood supply to the uterus, reduces the size of fibroids, resulting in decreased pain and dysmenorrhea with a high level of clinical improvement without the need of hysterectomy and better outcomes than myomectomy^(32)^. Uterine artery embolization has the advantage of managing the complications of myomectomy and other uterine procedures in a safe and effective way^(33)^. It may also deal with other causes of bleeding such as uterine artery pseudoaneurysm^(34)^, an underestimated clinical occurrence with many reports^(35,36,37)^, and 3.3% prevalence according to Dossou et al.^(5)^.

The importance of uterine artery embolization as well as the major role of the uterine artery in pregnancy and pelvic surgery marks the necessity for deep understanding of this vessel and its anatomic variations. Many studies described the origin of uterine arteries in different ways. Some of them used the Adachi classification on the iliac artery^(38)^, and some simply reported the vessel from which the artery originated. The characterization of this peculiar and significant artery requires specific classification because of the plethora of anatomic variations of uterine artery, and the different descriptions reported in the literature. In this review, the main origins of the uterine artery were the internal iliac artery, umbilical artery, and inferior gluteal artery. When the uterine artery comes from the internal iliac artery, it can branch separately or as bifurcation or trifurcation with other arteries, mainly the inferior and superior gluteal arteries. The internal pudendal artery is also reported to be the origin of the uterine artery in a notable percentage of cases. All these variations raise the challenge for a recognizable and accepted classification.

Uterine arteries play an important role in clinical practice. Internal iliac artery is described as their most common vascular origin. However, this review highlights that the main vessels of origin for uterine arteries are the internal iliac, umbilical, and inferior gluteal artery. Nevertheless, classification and further research for this peculiar anatomic structure is fundamental in the future.

## Figures and Tables

**Table 1:**
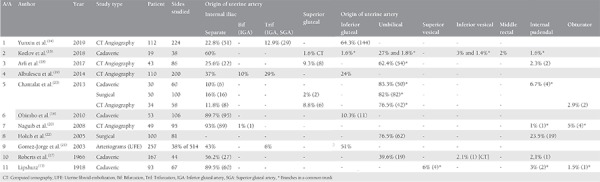
Origin of the uterine artery in the literature
